# The Mood, Mother and Child Study: Protocol for a Prospective Longitudinal Study and Randomized Controlled Trial

**DOI:** 10.2196/51132

**Published:** 2023-10-26

**Authors:** W Roger Mills-Koonce, Karen Grewen, Nisha Gottredson O’Shea, Brenda Pearson, Chelsea Grace Strange, Samantha E Meltzer-Brody, Jerry Dolph Guintivano, Alison M Stuebe

**Affiliations:** 1 School of Education The University of North Carolina at Chapel Hill Chapel Hill, NC United States; 2 School of Medicine The University of North Carolina at Chapel Hill Chapel Hill, NC United States; 3 Research Triangle Institute, Inc. Research Triangle Park, NC United States

**Keywords:** maternal depression, oxytocin, hypothalamic-pituitary-adrenal axis, HPA axis, parenting, executive functioning, socioemotional development

## Abstract

**Background:**

Perinatal depression affects >400,000 mother-child dyads in the United States every year and is associated with numerous adverse maternal and child developmental outcomes. Previous research implicates the dysregulation of oxytocin and the hypothalamic-pituitary-adrenal (HPA) axis functioning in mothers and children as potential mechanisms mediating or moderating the transmission of risk associated with maternal depression.

**Objective:**

The Mood, Mother and Child study will examine the psychobiological sources of risk and resilience within mother-child dyads affected by maternal depression. This manuscript describes (1) the study rationale and aims, (2) the research design and procedures and how they were altered in response to the COVID-19 pandemic, and (3) the data analysis plan to test the study hypotheses.

**Methods:**

This is a prospective longitudinal study with an embedded randomized controlled trial that examines (1) correlations among postpartum depression and anxiety symptoms, maternal and child oxytocin and HPA axis functioning, and child developmental outcomes and (2) the causal relationship between exogenous oxytocin and HPA reactivity. This study is funded by the National Institute of Child Health and Human Development with institutional review board approval.

**Results:**

Recruitment and data collection have commenced, and the expected results will be available in 2024. Analyses are presented for testing the proposed hypotheses.

**Conclusions:**

The unique combination of a prospective longitudinal research design with an embedded randomized controlled trial will allow the Mood, Mother and Child study to apply a developmental lens to the study of maternal depression and anxiety symptoms from birth to middle childhood and the psychobiological mechanisms promoting risk and resiliency for both mother and child outcomes. This will be the first study that simultaneously evaluates (1) the role of oxytocin using multiple methodologies, (2) the causal relationships between exogenous oxytocin and HPA axis functioning among mothers with differing levels of depression and anxiety symptoms, and (3) the multiple mediating and moderating roles of parenting behaviors and maternal and child psychobiological characteristics. The goals of these aims are to provide insights into the psychobiological effects of oxytocin in women and inform future clinical trials to treat perinatal mood disorders.

**Trial Registration:**

ClinicalTrials.gov NCT03593473; https://classic.clinicaltrials.gov/ct2/show/NCT03593473

**International Registered Report Identifier (IRRID):**

DERR1-10.2196/51132

## Introduction

### Background and Study Design Considerations

#### Overview

Perinatal depression (PND) is a common, morbid condition affecting >400,000 mother-child dyads in the United States each year [1]. In addition to the direct effect of PND on mothers’ health and well-being, it presents challenges for early child development, including reduced maternal sensitive caregiving [2]; impaired parent-child attachment relationships; and compromised child emotion regulation abilities [3,4], socioemotional development [5,6], and executive functioning (EF) [7,8]. These effects may occur even when mothers receive medication or psychotherapy for depressed mood [9], and they may extend well into adolescence and early adulthood [10,11]. Research has implicated the dysregulation of the oxytocin system [12] and the hypothalamic-pituitary-adrenal (HPA) axis [13] in the context of maternal depression as potential contributors to individual and parent-child relational dysfunction that increases the risk of nonoptimal child outcomes. The Mood, Mother and Child (MMC) study (funded by the National Institutes of Health; grant R01HD093901; grant review provided in Multimedia Appendix 1) addresses specific aims regarding the complex roles oxytocin functioning plays within the mother-child dyad, including the role of maternal oxytocin and HPA axis functioning as predictors of postpartum depression and anxiety symptoms (PDAS) and parenting behaviors and the role of child oxytocin functioning as both an outcome, mediator, and moderator of exposure to maternal depression and maladaptive parenting behaviors. This paper provides a brief synopsis of these goals and a detailed description of the MMC study protocol.

Both the severity and chronicity of maternal depression are associated with child developmental outcomes [14,15], and this association is (in part) mediated by dysregulated dyadic interactions between mother and child [16]. Symptomatic mothers are more likely to display withdrawn or intrusive behaviors when interacting with their infants in the first postpartum year [17,18], and these associations persist through early childhood, with the lowest levels of sensitive caregiving observed among mothers with chronic depression [19,20]. Building on previous research demonstrating the developmental risk associated with exposure to maternal depression, the MMC study will examine PDAS and associated parenting behaviors across early childhood as predictors of a variety of biopsychosocial outcomes in middle childhood, including EF, general cognitive development, and socioemotional adjustment. However, far less is known about the biopsychosocial processes underlying the manifestation of depressive symptoms in mothers and the transmission of risk in offspring. To address this gap, the MMC study will examine the roles of maternal and child oxytocin functioning, heart rate variability (HRV), and HPA axis functioning as potential sources of psychobiological risk and resilience.

#### Maternal Oxytocin Functioning, Depression, and Parenting

A subset of systems contributing to the biopsychosocial process underlying experiences of PND may include oxytocin and its effects on HPA functioning. Research suggests that PND is associated with dysregulation of the HPA axis [21] and that this association may (in part) be driven by dysregulated oxytocin functioning [22]. Previous studies on this topic—including research by the authors of this paper—have been mixed, including research demonstrating (1) no association between oxytocin during breastfeeding and PND [23], (2) an inverse association between oxytocin during breastfeeding and maternal symptoms of depression and anxiety at 2 months post partum [12], and (3) that oxytocin levels differentially predicted HPA axis reactivity in women with or without mood symptoms [13]. There are also candidate gene studies reporting correlations between oxytocin-related genetic variants and maternal depression [24] as well as oxytocin-related variants that predict developmental risk factors associated with maternal depression, such as stress reactivity [25,26], empathetic functioning [27], and parenting behaviors [28-30]. In addition, there is mixed evidence that exogenous administration of oxytocin can alter parenting behaviors [31-33] (for a systematic review, see the study by Szymanska et al [34]), although we are unaware of studies examining oxytocin administration and depressive symptoms. Furthermore, research on oxytocin and the psychobiology of PND has largely been conducted on mothers of infants and toddlers, with little empiricism on these topics in subsequent years of development. The MMC study will examine oxytocin functioning, oxytocin-related genetic variants, and HPA axis functioning as predictors of PDAS and parenting behaviors. This will be done using both a passive longitudinal design and a placebo-blinded trial examining the effects of exogenous administration of oxytocin on HPA axis functioning for mothers with and without depression diagnoses.

#### Child Oxytocin Functioning as a Potential Moderator and Mediator of PND

There is mixed evidence from candidate gene studies of oxytocin-related genetic variants functioning as moderators of the associations between adverse developmental exposures and subsequent child outcomes. For example, oxytocin receptor (OXTR) rs53576 G allele carriers who had experienced childhood maltreatment reported more depressive symptomatology compared with AA homozygotes, suggesting that the G allele confers increased vulnerability to stressors [35]; however, contradictory findings have reported that A allele carriage increased intergenerational transmission of maternal depression to child depressive symptomatology [36]. Meanwhile, a study of OXTR rs2254298 suggested that carriers of the A allele have increased developmental risk of anxiety and depression in the context of exposure to early-life adversity [37,38]. Finally, several studies indicate that these associations may vary by child sex [39,40] in the form of both sex-specific diathesis-stress and differential susceptibility models of gene × environment interaction. The MMC study will examine oxytocin-related candidate genes (as well as child sex) as potential moderators of child exposure to PDAS and maternal parenting behaviors with respect to a variety of outcomes in middle childhood, including EF and socioemotional adjustment.

In addition to the genetic-based moderation of exposure to PDAS and parenting behavior, there is evidence suggesting that epigenetic alterations in children’s oxytocin-related gene systems may mediate the associations between these exposures and child outcomes. Exposure to maternal depression is associated with differences in glucocorticoid receptor (NR3C1) methylation, which are thought to mediate associations between early-life adversity and subsequent psychopathology [41-45]. Furthermore, a recent genome-wide methylation study of children aged 1 year of mothers with or without major depressive disorder found differences across the epigenome [46]. It is also possible that the association between PDAS, maternal sensitivity, and child changes in epigenetic form may vary by child sex with respect to oxytocin functioning. Although speculative, research suggests that circulating oxytocin levels are comparable across boys and girls during the first 5 years of life [47,48]; however, oxytocin responsiveness to stress is significantly greater for girls who have a history of parental maltreatment as compared with girls without maltreatment or boys in either condition [49]. This alteration in functioning in girls may be a product of epigenetic changes in response to an extreme and maladaptive environment that accelerates reproductive development and individual fitness in the context of a dangerous environment; as such, if female individuals are genetically more sensitive to such environments, it is possible that markers of epigenetic change will be stronger for girls than for boys even at the age of 5 years. The MMC study will examine oxytocin-related changes in the epigenome from early infancy to middle childhood as potential mediators of associations between exposure to PDAS and parenting behavior and developmental outcomes, as well as the potential moderation of this pathway by child sex.

### MMC Study Aims and Hypotheses

The MMC study will address 3 specific aims. The first aim is to examine the role of oxytocin in the prediction of PDAS and other biopsychosocial maternal outcomes (eg, stress reactivity and parenting behaviors)—and specifically to examine the causal relationships between exogenous oxytocin and HPA reactivity. The following three hypotheses will be addressed as part of aim 1: (1) more severe PDAS will be associated with oxytocin and HPA axis dysregulation in correlational analyses, and causal relationships between oxytocin activity and HPA axis reactivity will be evidenced through a placebo-blinded trial of intranasal oxytocin effects on serum cortisol levels in response to a social challenge (hypothesis 1a); (2) genetic variation in maternal oxytocin-associated pathways will predict PDAS severity (hypothesis 1b); and (3) genetic variation will moderate associations among depression and anxiety symptoms, maternal oxytocin and HPA axis physiology, and parenting behaviors (hypothesis 1c).

The second aim is to examine the associations between PDAS and child biopsychosocial outcomes (eg, child epigenetics, EF, and cognitive and socioemotional development). The following four hypotheses will be addressed as part of aim 2: (1) more severe PDAS will predict more adverse child biopsychosocial outcomes during early elementary school years (hypothesis 2a); (2) high stress reactivity and maladaptive parenting behaviors will mediate associations between more severe PDAS experienced during the first year post partum and adverse child biopsychosocial outcomes in years 5 to 7 (hypothesis 2b); (3) the direct and indirect effects of PDAS on externalizing behaviors at ages 5 to 7 years will be stronger for boys, the direct and indirect effects of PDAS on internalizing behaviors at ages 5 to 7 years will be stronger for girls, and the direct and indirect effects of PDAS on early elementary cognitive outcomes will be comparable across boys and girls (hypothesis 2c); and (4) the effects of PDAS on early childhood outcomes will persist even after accounting for concurrent maternal depression and anxiety symptoms (hypothesis 2d).

The third aim is to examine the associations from the previous aim as moderated by child oxytocin-related genotype and mediated by child oxytocin-related epigenetic changes over time. The following five hypotheses will be addressed as part of aim 3: (1) child genetic variation in oxytocin-associated pathways will moderate the associations between PDAS and child biopsychosocial outcomes (hypothesis 3a); (2) child genetic variation in oxytocin-associated pathways will moderate the associations between maternal parenting behaviors and child biopsychosocial outcomes (hypothesis 3b); (3) child sex will moderate relationships among child oxytocin genetic variation, PDAS, parenting behaviors, and child biopsychosocial outcomes (hypothesis 3c); (4) child epigenetic changes underlying oxytocin functioning between the ages of 2 months and 5 years will mediate the associations between PDAS exposure and parenting behaviors and child biopsychosocial outcomes (hypothesis 3d); and (5) the associations between PDAS or parenting behaviors and child epigenetic changes will be stronger for girls than for boys (hypothesis 3e [exploratory hypothesis]).

## Methods

### Overview

The MMC study recruits mother-child dyads who previously participated in the Mood, Mother and Infant (MMI) study [50]. Although MMC study data collection focuses on mothers and children during the middle childhood period (age of approximately 6 y), using this existing sample will provide for longitudinal analyses spanning from the prenatal period to the current age of the child. Participation involves completing 2 laboratory-based data collection visits along with web-based surveys completed outside the visit (typically at home before the final visit). The first visit involves data collection using a double-blinded randomized experimental design and involves only the biological mother. During this visit, nonpregnant mothers are randomized to either the nasal oxytocin or placebo condition (based on a randomization table created using a random number generator from the University of North Carolina at Chapel Hill investigational drug service). The second laboratory visit involves data collection with mothers and children for the purpose of hypothesis testing within the passive longitudinal design.

Data collection began on February 7, 2019, and was scheduled to continue until the spring of 2022. However, data collection was interrupted by the COVID-19 pandemic. On March 14, 2020, data collection was halted entirely and did not resume until August 25, 2020, at which point an adapted protocol was implemented for the safety of participant families and research staff. On April 21, 2021, the original protocol was largely reinstated and will be implemented until the completion of the project (participants continued to wear masks beyond this date, and the order of the child data collection protocol remained the same as during the pandemic). Descriptions of the data collection protocols for the pre– and post–COVID-19 periods and the protocol during the COVID-19 period are provided in the following sections.

### Participants

Participants for the MMC study are recruited from the MMI sample (N=222). Table 1 provides a summary of descriptive information for this sample. Mothers in the MMI study were initially recruited in the third trimester of pregnancy and had a singleton pregnancy, and all reported intention to breastfeed (this was relevant because of the centrality of breastfeeding to the oxytocin assessments and hypotheses in the MMI study). The sample was enriched with women at a high risk of depression or anxiety such that 87 (39.2%) had a Structured Clinical Interview for the Diagnostic and Statistical Manual of Mental Disorders, Fourth Edition (DSM-IV)–verified current diagnosis of depression or anxiety or were in active treatment with psychotherapy or antidepressants, 64 (28.8%) had a past diagnosis of depression or anxiety, and 71 (32%) were low risk with no history of depression or anxiety. After completing the final in-person data collection visit at 12 months of child age, mothers were invited to participate in web-based follow-up questionnaires every 6 months until the child was aged 4 years. Of these mothers, 92% (138/150) chose to participate in ongoing data collection, leading to a projected enrollment of 150 dyads for the MMC study. Owing to additional attrition and suspension of data collection because of the COVID-19 pandemic, the MMC enrollment target has been reduced to 110 dyads.

**Table 1:** Demographic information on the original Mood, Mother and Infant study.

			Depression diagnosis				
			Current (n=71)		Previous but not current (n=64)		Never (n=87)
Primiparous			37 (52)		22 (34)		40 (46)
**Race or ethnicity**							
	Asian	5 (7)		3 (5)		4 (5)	
	Black	11 (15)		9 (14)		8 (9)	
	Hispanic or Latino	9 (13)		6 (9)		7 (8)	
	American Indian or Alaska Native	1 (1)		1 (1)		3 (4)	
	White	59 (83)		52 (81)		76 (87)	
Married			59 (83)		52 (81)		66 (76)
**Education**							
	<4 y of college	12 (17)		17 (26)		26 (30)	
	4 y of college	27 (38)		21 (33)		18 (21)	
	Postgraduate	32 (45)		26 (41)		43 (49)	

### Procedures

#### Visit 1: The Randomized Controlled Trial Administration

##### Pre– and Post–COVID-19 Protocol

Following the mother’s arrival and informed consent procedures, each participant provides a urine sample used immediately for pregnancy testing, and only nonpregnant participants proceed with the oxytocin randomized controlled trial (RCT) visit. Research staff apply MindWare cardiovascular sensors (MindWare Technologies LTD) to monitor HRV and the cardiac pre-ejection period (PEP), a SunTech Tango monitor (SunTech Medical, Inc) assesses blood pressure and heart rate, and an antecubital intravenous catheter is placed for interval blood sampling. Next, the mother is left to adapt to the testing chamber for a 10-minute habituation period [51,52]. Following the habituation period [53-56], participants are randomized by risk level in a 1:1 allocation to either 24 international units of nasal oxytocin or a placebo nasal spray in an identical bottle. Both participants and study team members are blinded to allocation. The study drug and placebo are obtained from Apotheke Roter Ochsen. A total of 40 minutes after the nasal spray administration, mothers participate in the Trier Social Stress Test (TSST). The TSST includes a speech performance task and a math performance task, which reliably induce large and consistent HPA and cardiovascular responses [52,57]. Following the guidelines for examining HPA stress reactivity, intravenous blood samples are collected at baseline; at 20 and 40 minutes of the post–nasal spray rest period; during the speech and math tasks; and at 10, 20, and 30 minutes of the post-TSST recovery period [52,58]. Across these 8 samples, a total blood volume of 113 mL is collected. Each maternal blood sample is collected into prechilled vacutainer tubes, immediately cold centrifuged, aliquoted into prechilled cryotubes, and stored at −80 °C. Owing to conflicting evidence regarding the optimal timing of stress testing [59,60], visits are scheduled for 1 PM to increase the likelihood of detecting a stress response unopposed by the circadian influence [22]. Previous findings suggest that the possible menstrual cycle phase does not affect TSST results [61,62]; therefore, although the date of the last menstrual period and hormones are not used for scheduling data collection visits, these data are recorded for analytic control purposes. Staff also conduct the nonpatient version of the Structured Clinical Interview for the DSM-IV (SCID-NP), to assess the mother’s current psychiatric symptoms [52] at this visit.

##### Protocol Amendments Owing to COVID-19

During the months affected by the COVID-19 pandemic, the MMC study visit 1 protocol was amended as follows. First, participants completed informed consent protocols over the phone using DocuSign (DocuSign, Inc). The research staff reviewed a COVID-19 information sheet and symptom screening before arriving at the study visit. Participants were also screened for symptoms upon arrival. Second, the SCID-NP was conducted via phone call instead of in person. Finally, research staff and participants further enhanced safety protocols by requiring masking for all, limiting contact by leaving the testing chamber when possible, keeping the door open for increased air circulation, and remaining a minimum of 6 feet away as much as possible. The TSST committee members were seated >6 feet away from the participant and from each other. These protocol amendments will be implemented for the remainder of the study.

#### Visit 2: Mother and Child Data Collection for Hypothesis Testing

##### Pre–COVID-19 Protocol

Following the mother’s arrival and informed consent procedures, MindWare cardiovascular sensors were placed on the mother and child, 6 cheek cell swabs were collected from the child, and the child’s height and weight were recorded. After a 5-minute habituation period to the testing chamber, the mothers were taken into a neighboring room, and the children remained in the assessment room with the research staff. The children were then assessed for EF ability using the EF Touch assessment (a computerized EF battery validated for use in children aged 4 to 7 y). This lasted for approximately 30 to 45 minutes (including practice, administration, and breaks between tasks). Finally, child IQ was assessed using the 6 subscales of the Wechsler Preschool and Primary Scale of Intelligence that comprise the full-scale IQ (FSIQ) measure [56]. While the children completed the EF and IQ assessments, the mothers were asked to complete a series of computer-based EF assessments from Inquisit, including the Wisconsin Card Sorting Test, Visual Simon Task, Symbol Search, Simple Visual Reaction Time Task, Self-Ordered Pointing Task, Digit Symbol Substitution Task, Counting Span Task, Stroop Task, and Tower of London Task, which took approximately 45 minutes. Mothers then completed the Child Behavior Checklist (CBCL) and a recorded speech for which they were instructed to talk for 5 minutes about their child.

Once the mothers and children were done with their separate tasks, a maternal antecubital intravenous was placed for interval blood sampling. The dyad was then reunited for an additional 5-minute baseline rest period. After this period, the mother and child participated in a set of 3 parent-child interaction tasks designed to assess parent and child behaviors within a dynamic interaction context [63]. Three 5- to 10-minute tasks were used in this interaction. The first was a tower-building task in which the dyad was instructed to recreate a large wooden block tower using smaller, differently shaped wooden blocks. The dyad was instructed that the task was for the child to complete but the mother could provide any assistance she chose. The second task involved a simple card game with a standard deck of playing cards divided evenly between the mother and child. Each was instructed to simultaneously turn over a card and place it face up in the space between them. This continued until one of the cards was a jack, at which point the first person to slap the jack card won all the cards that had accrued to that point. The goal of the game was to win as many cards as possible. For the third interaction task, the dyad was provided with an Etch A Sketch toy (Ohio Art Company) and a line picture of a sailboat. Mothers were instructed to use one of the Etch A Sketch knobs, and the children were instructed to use the other; their goal was to recreate the picture of the sailboat on the Etch A Sketch by working cooperatively with each using a separate turning knob on the toy. These parent-child interaction tasks were recorded for later behavioral coding. Maternal blood was collected before and immediately after the parent-child interaction tasks; a total blood volume of 59 mL was collected. Each maternal blood sample was collected into prechilled vacutainer tubes, immediately cold centrifuged, aliquoted into prechilled cryotubes, and stored at −80 °C. Once participants were deinstrumented, research staff collected hair samples from the mother and child for cortisol analysis.

##### Post–COVID-19 Protocol

During the months affected by the COVID-19 pandemic, the MMC study visit 2 protocol was amended as follows. First, we suspended maternal blood sampling, mother and child MindWare data and hair sample collection, and child cheek cell collection to reduce contact between staff and participants. Second, upon arrival (after completion of wellness screening), mothers and children were directed into a cleaned, unoccupied room without research staff where they could remove their masks to complete the parent-child interaction tasks (cameras were operated remotely for recording). These safety accommodations were important as it was necessary to observe these interactions without masks to code mother and child facial affect. Once the parent-child interaction tasks were complete, mothers and children resumed mask wearing. Suspended protocols were gradually reintroduced as COVID-19 restrictions were reduced. On April 21, 2021, cheek cell collection resumed. On August 23, 2021, MindWare data collection for the children and the baseline rest period were reintroduced. On June 26, 2022, hair samples were reintroduced and moved to the beginning of the protocol. Maternal blood collection and MindWare data collection were not implemented for the remainder of the study. Owing to this, the second baseline rest period was never reintroduced, either. The research staff reviewed a COVID-19 information sheet and symptom screening before arriving at the study visit. Participants were also screened for symptoms upon arrival. Research staff and participants increased safety protocols, including required masking for all, limiting contact by leaving the chamber when possible, keeping the door open for increased air circulation, and remaining a minimum of 6 feet away as much as possible. These protocols will be implemented for the remainder of the study.

### Measures

#### Overview

**Figure 1:**
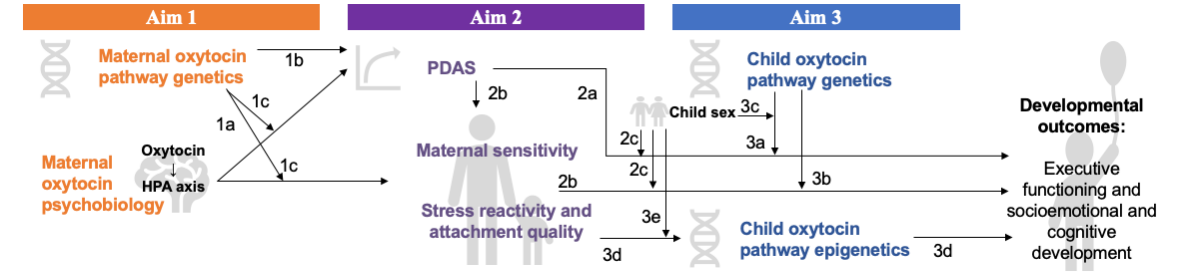
Conceptual model for the Mood, Mother and Child Study. HPA: hypothalamic-pituitary-adrenal; PDAS: postpartum depression and anxiety symptoms.

MMC study measures are presented in the following sequence. First, measures pertinent to the RCT examining oxytocin and HPA axis functioning are presented. Next, measures pertinent to testing the hypotheses in aims 1, 2, and 3 are presented in order of appearance in the passive longitudinal design (Figure 1). This begins with maternal oxytocin functioning and oxytocin genetics as primary predictors (maternal oxytocin psychobiology and oxytocin genetics) followed by maternal biopsychosocial outcomes (PDAS and maternal parenting behaviors) and then child biopsychosocial outcomes (child oxytocin epigenetics, stress reactivity, and cognitive and socioemotional outcomes). Summaries of potential moderators and control variables are also provided.

#### RCT Measures

##### Independent Variable: Intranasal Oxytocin Administration

Mothers in the experimental condition are administered 24 international units of nasal oxytocin, and mothers in the placebo condition are administered an equivalent volume of an inert nasal spray. After a rest period, mothers are trained and observed to self-administer 6 separate nasal spray inhalations at approximately 30- to 60-second intervals, alternating between the right and left nostril.

##### Dependent Variable: HPA Axis Functioning

Maternal blood samples from the first laboratory visit (including baseline; during the TSST speech and math tasks; and at 10, 20, and 30 min of the recovery period following the TSST) will be assayed for adrenocorticotropic hormone in ethylenediaminetetraacetic acid–treated plasma and serum cortisol using commercially available radioimmunoassay reagents from MP Biomedicals.

##### Primary Predictors: Maternal Oxytocin Functioning

###### Maternal Oxytocin Secretion

Oxytocin in ethylenediaminetetraacetic acid–treated plasma, with aprotinin added to prevent peptide degradation, will be determined through enzyme immunoassay with extraction (Enzo Life Sciences), as previously described [64]. Interpretation of the oxytocin literature is complicated by controversies in the assay technique [65] as many studies report results for unextracted samples. These results are difficult to interpret given that Szeto et al [66] found no correlation between the extracted and unextracted oxytocin values. A key strength of our study is that we will use the extracted oxytocin for all measures.

###### Maternal Oxytocin Genetics

DNA extracted from maternal blood collected during the MMI study will be used for genotyping. Genome-wide single nucleotide polymorphism (SNP) arrays will be used to identify genetic variations in individual genes (in addition to clusters of genes) whose products interact in pathways that have been implicated in maternal depression, stress reactivity, and parenting behaviors (such as oxytocin). To estimate genetic ancestry in our diverse population, we will apply both structure and Eigensoft SmartPCA to linkage equilibrium-pruned array data.

##### Maternal Biopsychosocial Outcomes

###### Maternal Depression and Anxiety

Depression and anxiety symptoms are measured at multiple times (Table 2), including the SCID-NP [53] in the third trimester of pregnancy (for the MMI study) and again at the first data collection visit at 5 years of child age in the MMC study. The Structured Clinical Interview for the DSM-IV is a semistructured interview for assessment of major DSM-IV axis-I diagnoses and is considered the gold standard instrument for rigorous assessment of lifetime and current psychiatric illness (the nonpatient version was specifically developed for use in research studies). In addition, monthly assessments were conducted during the first year of life using the Edinburgh Postnatal Depression Scale [67]; assessments at 2, 6, and 12 months were conducted using the Beck Depression Inventory–II [68] and the Spielberger State-Trait Anxiety Inventory [69]; and all 3 measures were collected every 6 months thereafter from the ages of 1 to 4 years and then again at the current MMC study visit.

**Table 2:** Maternal depression and anxiety measurement over time.

	Prenatal—third trimester	Year 1 (month)													Year 2 (month)			Year 3 (month)			Year 4 (month)			Years 5-7—month 60 onward
		1	2	3	4	5	6	7	8	9	10	11	12	18		24	30		36	42		48		
SCID^a^	✓																						✓	
EPDS^b^	✓	✓	✓	✓	✓	✓	✓	✓	✓	✓	✓	✓	✓	✓		✓	✓		✓	✓		✓	✓	
BDI^c^	✓		✓				✓						✓	✓		✓	✓		✓	✓		✓	✓	
SSA^d^	✓		✓				✓						✓	✓		✓	✓		✓	✓		✓	✓	

^a^SCID: Structured Clinical Interview for the Diagnostic and Statistical Manual of Mental Disorders, Fourth Edition.

^b^EPDS: Edinburgh Postnatal Depression Scale.

^c^BDI: Beck Depression Inventory.

^d^SSA: Spielberg State-Trait Anxiety Inventory.

###### HRV Measure

Mobile Impedance Cardiographs (MindWare Technologies LTD) are used to measure cardiac rate, interbeat interval, and PEP in mothers and children. MindWare HRV software will be used to derive respiration and calculate high-frequency HRV and respiratory sinus arrhythmia from the interbeat interval series as indexes of parasympathetic activity. Respiratory rate will be used in all HRV analyses. MindWare Impedance Cardiography Analysis software will calculate cardiac time intervals, stroke volume, and cardiac output. PEP will index sympathetic activation.

###### Chronic HPA Activation

Hair cortisol will be assayed from 20 to 25 strands of hair [70] collected from mothers and children using a commercially available immunoassay with chemiluminescence detection (IBL-Hamburg) that has been validated using liquid chromatography mass spectrometry.

###### Parenting Behaviors

Video recordings of parent-child interactions collected at visit 2 will be coded by a team of trained and certified researchers led by WRM-K. Ratings of maternal sensitivity, detachment, intrusiveness, positive regard, negative regard, stimulation of cognitive development, and animation will be made using a 5-point Likert scale (ranging from 1 as *not at all characteristic* to 5 as *highly characteristic*). On the basis of previous empirical findings and theoretical models of parenting dimensions, we anticipate that these scales will load onto 2 broad dimensions of maternal parenting behavior—*overall sensitivity* (comprising sensitivity, detachment [reversed], stimulation, positive regard, and animation) and *negative-controlling behaviors* (negative regard and intrusiveness). The ultimate creation of parenting dimensions will be guided by factor analyses comparable with those in previous studies by WRM-K [71].

##### Child Biopsychosocial Outcomes

###### Child Epigenetics

DNA extracted from child cheek swabs at the ages of 2 months and 5 years will be used to assess genome-wide DNA methylation using the Illumina MethylationEPIC BeadChip. This platform interrogates 850,000 methylation sites across the genome at single-base resolution; target gene regulatory regions include enhancers, CpG islands, promoters, and open chromatin sites. Methylation probes are dropped from the analysis for bad genome mapping, missingness (>0.01), and low detection (<0.05). Raw microarray signal intensity (SI) data are first corrected using the Illumina probe type, followed by individual methyl and nonmethyl channel quantile normalization using the *limma* package in Bioconductor. The methylation status of each CpG site is then calculated as the β value based on the following definition: β value = (SI of the methylation detection probe) / (SI of methylation detection probe + SI of non–methylation detection probe + 100). Cell type composition will be estimated as a covariate for association analyses using the *minfi* package in Bioconductor. Interindividual DNA methylation trajectories will be identified from the age of 2 months to 5 years. Although all relevant methylation sites will be examined, particular emphasis will be placed on oxytocin pathways and their effects.

###### Child EF

Children’s EF is assessed at visit 2 via performance on the EF Touch instrument, which is a computerized battery of EF tasks using a capacitive touch screen monitor to record child responses [72]. Working memory is assessed using the Working Memory Span Task [73] and the Pick the Picture Game [74,75]; inhibitory control is assessed using the Silly Sounds Stroop [76], Spatial Conflict Task [77], and animal Go or No-Go Task [78]; and attention shifting is assessed using the Something’s the Same Game [79]. Each of these 5 tasks takes 3 to 7 minutes to complete. For complete descriptions of these tasks and extensive psychometric and reliability information, see the study by Willoughby et al [80].

###### Child IQ

Child IQ was assessed at visit 2 using 6 subscales of the Wechsler Preschool and Primary Scale of Intelligence that comprise the FSIQ composite. These include the Block Design (assessing spatial reasoning), Information (assessing cultural knowledge), Matrix Reasoning (assessing visual processing), Bug Search (assessing speed of processing), Picture Memory (assessing working memory), and Similarities (assessing logical thinking) subscales. The FSIQ composite is considered the most reliable and representative measure of broad cognitive functioning in middle childhood [81].

###### Child Maladaptive Behaviors

Mothers completed the CBCL for school-age children [82]. The CBCL is a highly used instrument that assesses emotional and behavioral problems in children and produces broad-band (internalizing problems, externalizing problems, and total problems) and narrow-band (eg, attention problems, sleep problems, anxiety or depression, and aggressive behavior) scales with standardized T scores.

##### Potential Moderating Variables

###### Child Sex

Maternal report of child sex at birth will be used as a moderating variable in hypotheses 2c, 3c, and 3e.

###### Child Oxytocin Genetics

DNA extracted from child cheek swabs will be used for genome-wide SNP arrays to identify (1) aggregate genetic risk (eg, genetic risk scores for psychiatric outcomes) and (2) genetic variation of individual genes (in addition to clusters of genes) associated with oxytocin functioning; these data will be used as moderating variables in hypotheses 3a and 3b.

##### Potential Control Variables

In addition to demographic controls (eg, maternal or child race and ethnicity and maternal education, age, and family income), additional control variables will be collected and used as appropriate for specific hypothesis testing. For example, analyses of child EF and IQ outcomes will include control variables for maternal EF [55]. Child temperament (assessed using the Children’s Behavior Questionnaire [83]) will be used to control for genetic confounds between mothers and children as well as passive gene-environment correlations within the family system. Current psychiatric treatment for maternal depression and anxiety (both pharmacological and nonpharmacological) will be included as appropriate.

### Data Analysis Plan

#### Characterizing PDAS

**Figure 2:**
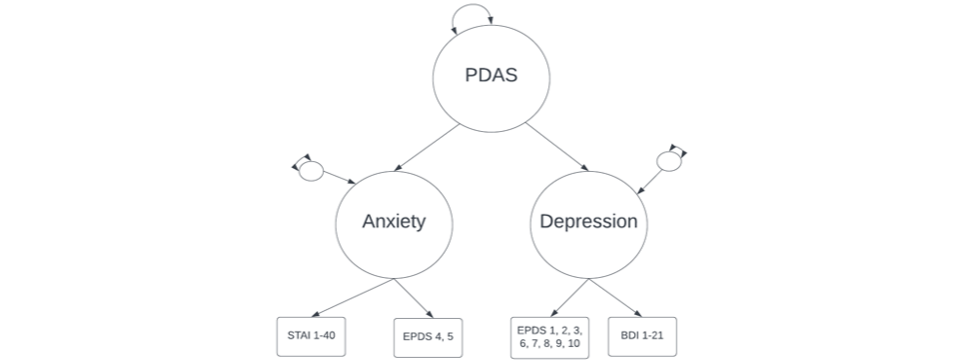
Hierarchical confirmatory factor analysis model of postpartum depression and anxiety symptoms (PDAS) factor. Numbers reflect child age (in months) at time of assessment. BDI: Beck Depression Inventory; EPDS: Edinburgh Postnatal Depression Scale; STAI: Spielberg State-Trait Anxiety Inventory.

We plan to integrate PDAS measures obtained from repeated assessments of the Edinburgh Postnatal Depression Scale, Beck Depression Inventory–II, and State-Trait Anxiety Inventory by constructing a hierarchical confirmatory factor analysis (HCFA) model with a general PDAS factor giving rise to anxiety and depression subdomains (Figure 2). We will incorporate orthogonal method factors (not shown in the figure) to account for the scale of origin. The HCFA model will be estimated using 1 randomly drawn observation from each mother’s first year of postpartum data. Once we have obtained a good-fitting HCFA model, we will use the parameter estimates from the calibration model to generate time-varying maternal PDAS factor score estimates—1 per month during the first year post partum [84].

Once time-varying PDAS scores have been obtained, we will determine the optimal method for characterizing maternal PDAS. We will consider three approaches: (1) latent mean, (2) growth parameters, and (3) growth mixture classes. We describe each of these approaches in the following paragraphs.

First, we will generate spaghetti plots to examine the shape and heterogeneity of PDAS scores over time. We will use linear mixed models to construct univariate growth models for PDAS to determine empirically whether scores follow a linear, quadratic, or cubic average trajectory and whether there is significant individual heterogeneity in change over time. The following set of equations presents an example of a quadratic growth trajectory for PDAS, where *i*=1...N indexes individuals and *t*=0...12 indexes time in months, starting immediately post partum. Time is centered at 6 months post partum in the equation. The *b* parameters are the fixed effects that characterize the PDAS trajectory for the average participant. The *u* parameters are individually varying random effects that define each participant’s unique PDAS trajectory. We assume that the *u* terms follow a multivariate normal distribution centered at 0 and that the residual error is normally distributed at each time point. The residual error is assumed to be uncorrelated with the random effects.

PDAS*_ti_* = *b*_0_ + *b*_1_ × (*t* − 6) + *b*_2_ × (*t* − 6)^2^ + *u*_0_*_i_* + *u*_1_*_i_* × (*t* − 6) + *u*_2_*_i_* × (t − 6)^2^ + *r*_t_*_i_*



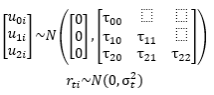



**(1)**

If we do not find a significant fixed effect of time (ie, if *b*_1_ and *b*_2_ are not significantly different from 0), we will output empirical Bayes estimates of the random intercept and use these scores to represent each participant’s average PDAS score 
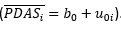
 . We refer to this first approach as the latent mean approach.

If individual trajectories follow the same general form (eg, all linear but with continuously varying slope values), we will output empirical Bayes estimates of mother-specific intercepts (*int*_i_ = *b*_0_ + *u*_0_*_i_,* representing the average PDAS at 6 mo post partum) *and* slopes (*slope*_i_ = *b*_1_ + *u*_1_*_i_*, representing the direction and rate of change over time). In this “growth parameter” approach, we will represent PDAS trajectories using a combination of 2 or more growth parameters.

Finally, if we find visual and empirical evidence of substantial heterogeneity in the *shape* of PDAS change over time, we will use growth mixture models to estimate a small number of prototypical PDAS trajectory shapes and then use posterior probability scores to assign participants probabilistically to a trajectory class. This third approach will allow for a small number of different trajectories (eg, 1 stable trajectory, 1 increasing linearly, and 1 decreasing to a plateau). Each participant will receive a posterior probability of membership to each trajectory class. Once PDAS trajectories have been operationalized as (1) latent means, (2) growth parameters, or (3) growth mixture classes, we will use the resulting measures to address the study hypotheses.

#### Aim 1 Hypothesis Testing

First, we will use a general linear model to test the hypothesis that participants who are randomized to receive nasal oxytocin experience lower HPA reactivity during the TSST to evaluate this proposed causal pathway (hypothesis 1a). Next, we will also evaluate whether observed oxytocin functioning or genetic variation in oxytocin-associated pathways correlates with PDAS and will then use structural equation modeling (SEM) to assess whether mothers’ HPA functioning mediates the association between oxytocin variation and PDAS (hypotheses 1a and 1b). The SEM framework will enable us to model uncertainty in the growth parameters or latent class membership while simultaneously estimating the 2 mediation pathways (oxytocin to maternal HPA functioning [*a* path] and maternal HPA functioning to PDAS [*b* path]). We will use bootstrapped SEs to conduct significance tests for the indirect effects.

Finally, multigroup SEM will be used to examine the potential of moderated mediation, with maternal genetic variation in oxytocin-related pathways moderating the associations between maternal oxytocin and HPA functioning and PDAS (hypothesis 1b). We will also test whether maternal genetic variation moderates the associations between PDAS and maternal HPA functioning, oxytocin functioning, and parenting behaviors (hypothesis 1c). Significant moderation effects will be probed following procedures outlined by Preacher et al [85] to obtain estimated effect conditions on genetic variation within the range of our data.

#### Aim 2 Hypothesis Testing

Our next step of analysis will be to evaluate the extent to which PDAS predicts more adverse child biopsychosocial outcomes during early elementary years (hypothesis 2a), whether high stress reactivity or maladaptive parenting behaviors mediate this association (hypothesis 2b), whether the direct effects of PDAS on child outcomes remain significant after accounting for concurrent maternal depression and anxiety severity (hypothesis 2b), and whether sex moderates the associations between PDAS and child outcomes such that the effects of PDAS on child externalizing are stronger for male individuals and the effects of PDAS on child internalizing are stronger for female individuals. We will continue within an SEM framework for aim 2 analyses as this approach allows for the simultaneous modeling of mediation, moderation, and a variety of response distributions.

In addition to demographic control variables, we will include child temperament (assessed in infancy and concurrently at the age of 5 y) to control for genetic confounds between mothers and children as well as passive gene-environment correlations within the family system.

#### Aim 3 Hypothesis Testing

For aim 3, we will conduct SEM analyses in two ways: (1) including estimated risk scores and (2) stratified by child OXT and OXTR genotypes to determine if child genotype moderates the associations between PDAS and developmental outcomes (hypothesis 3a) and between maternal sensitivity and developmental outcomes (hypothesis 3b). Next, we will test whether the pathways tested in aims 1 and 2 vary by child sex by introducing 2-way interactions between PDAS and child sex and parental sensitivity and child sex and probing significant effects. We will test whether the methylation status of oxytocin-related genetic regions mediates associations among PDAS exposure, sensitivity, attachment quality, and developmental outcomes (hypothesis 3d). Finally, we will conduct exploratory analyses using multigroup models to examine whether the effects of PDAS and maternal sensitivity on epigenetic changes in child oxytocin functioning are stronger for girls than for boys (hypothesis 3e). We will use bootstrapped SEs to conduct significance tests for the indirect effects. Moderation effects will be probed following the procedures outlined by Preacher et al [85].

#### Missing Data

The proposed study design incorporates extensive repeated assessments. Growth models and SEMs are estimated using full information maximum likelihood, which permits the assumption that missing data are missing at random conditional on observed measures. We will rely on this assumption when data are missing exclusively on endogenous variables. We will use multiple imputation for all analyses involving missing covariates to avoid listwise deletion of cases with missing exogenous variables.

#### Power Analysis

As described previously, attrition and suspension of data collection because of COVID-19 resulted in a lower enrollment in the MMC study than we had hoped; however, we anticipate enrolling 110 dyads from the original sample. With this sample size and a type-I error rate of Cronbach α=.05, we will have 80% power to detect a correlation of *r*≥0.26, a mean difference of Cohen *d*≥0.5, and an odds ratio of ≥2.02. Thus, broadly, we expect to have the power to detect moderate, clinically meaningful effects; however, we will have limited power to detect smaller effect sizes that tend to occur in mediation and moderation analyses.

### Ethics Approval

This project received ethics approval from the University of North Carolina at Chapel Hill institutional review board (21-0421).

## Results

The trial was registered with ClinicalTrials.gov on July 20, 2018 (NCT03593473). The study began in September 2018 and continues through August 2024. Recruitment and data collection have commenced; as of June 1, 2023, a total of 102 participants have been enrolled. Study results should be available in 2024.

## Discussion

Previous research has documented associations between PND and offspring developmental outcomes in middle childhood [30,86] and evidenced the role of oxytocin in PND [87]. However, no study has evaluated the role of oxytocin using multiple methodologies (eg, circulating oxytocin, exogenous oxytocin administration, SNP, and epigenetic analyses) in mothers and children using a longitudinal design spanning infancy to middle childhood. Our unique combination of methodologies will allow for potential attributions of causality with respect to oxytocin administration while allowing us to unpack the psychobiological processes underlying these effects with the ongoing longitudinal design. These results will provide unique insights into the psychobiological effects of oxytocin in women and may inform future clinical trials to treat perinatal mood disorders [88]. Furthermore, we will be the first to report on interindividual measures of oxytocin-related DNA methylation in children as a function of maternal depression and parenting experiences [89]. Previous studies have approached these topics in a piecemeal fashion within time and across single levels of analysis, whereas our integrated, multilevel, multi-method longitudinal approach will elucidate the role of oxytocin in dyadic development and identify psychosocial, genetic, and epigenetic markers that can be leveraged to cultivate resilience across 2 generations.

## Appendix

Multimedia Appendix 1 Final National Institutes of Health grant proposal review.

## Abbreviations

CBCL: Child Behavior Checklist
DSM-IV: Diagnostic and Statistical Manual of Mental Disorders, Fourth Edition
EF: executive functioning
FSIQ: full-scale IQ
HCFA: hierarchical confirmatory factor analysis
HPA: hypothalamic-pituitary-adrenal
HRV: heart rate variability
MMC: Mood, Mother and Child
MMI: Mood, Mother and Infant
OXTR: oxytocin receptor
PDAS: postpartum depression and anxiety symptoms
PEP: pre-ejection period
PND: perinatal depression
RCT: randomized controlled trial
SCID-NP: nonpatient version of the Structured Clinical Interview for the Diagnostic and Statistical Manual of Mental Disorders, Fourth Edition
SEM: structural equation modeling
SI: signal intensity
SNP: single nucleotide polymorphism
TSST: Trier Social Stress Test

## Data Availability

The data sets generated from this study are not publicly available because of ongoing data collection protocols but will be available at the conclusion of this study from the corresponding author upon reasonable request following guidelines stipulated by the National Institutes of Health.
