# Supplementary material for: The Mood, Mother and Child Study: Protocol for a Prospective Longitudinal Study and Randomized Controlled Trial
Source: JMIR Res Protoc. 2023 Oct 26;12:e51132. doi: 10.2196/51132 (PMC10636628; doi:10.2196/51132)
Supplement: Multimedia Appendix 1 [file resprot_v12i1e51132_app1.pdf]

SUMMARY STATEMENT
(Privileged Communication)

PROGRAM CONTACT:
LAYLA ESPOSITO
(301) 435-6888
espositl@mail.nih.gov

Release Date: **03/09/2018**
Revised Date:

Application Number: **1 R01 HD093901-01A1**

Principal Investigators (Listed Alphabetically):

MILLS-KOONCE, WILLIAM ROGER
STUEBE, ALISON M (Contact)

Applicant Organization: UNIV OF NORTH CAROLINA CHAPEL HILL

Review Group: **MESH**
Biobehavioral Mechanisms of Emotion, Stress and Health Study Section

Meeting Date: **02/26/2018**
Council: **MAY 2018**
Requested Start: **07/01/2018**
RFA/PA: **PA16-160**
PCC: **CDBB -LE**
Dual IC(s): **MH**

Project Title: **Mood, mother, and child: The psychobiology of dyadic resilience**

SRG Action: **Impact Score:26 Percentile:10**
Next Steps: **Visit https://grants.nih.gov/grants/next_steps.htm**
Human Subjects: 30-Human subjects involved - Certified, no SRG concerns
Animal Subjects: 10-No live vertebrate animals involved for competing appl.
Gender: 1A-Both genders, scientifically acceptable
Minority: 1A-Minorities and non-minorities, scientifically acceptable
Children: 1A-Both Children and Adults, scientifically acceptable
Clinical Research - not NIH-defined Phase III Trial

Project Year	Direct Costs Requested	Estimated Total Cost
1	499,999	769,478
2	499,999	769,478
3	499,999	769,478
4	499,999	769,478
5	499,999	769,478
TOTAL	2,499,995	3,847,388

ADMINISTRATIVE BUDGET NOTE: The budget shown is the requested budget and has not been adjusted to reflect any recommendations made by reviewers. If an award is planned, the costs will be calculated by Institute grants management staff based on the recommendations outlined below in the COMMITTEE BUDGET RECOMMENDATIONS section.

1R01HD093901-01A1 Stuebe, Alison

RESUME AND SUMMARY OF DISCUSSION: Building on an existing cohort of mother-infant dyads, the proposed longitudinal study will examine maternal depression and infant outcomes in order to identify mediators and moderators of infant risk and resilience. If successful, the study could identify biological, psychological, and mother-child behavior targets for mental health prevention and intervention. The focus on mother-child interaction is significant and appropriate. The premise that oxytocin is a principal linchpin in dyadic development is strong. The revision is responsive to prior review and as a result is considerably strengthened. Other strengths include leveraging an existing study cohort and data, the innovative oxytocin challenge, overall highly rigorous methods including multilevel psychobiological assessment of oxytocin and peptides, and a sophisticated analytic strategy. The investigative team is superb and well-qualified to successfully carry out the study; feasibility is deemed high due to supportive preliminary data and extensive history of collaboration in this setting. There is thoughtful consideration of effects of child sex as a biological variable. A few minor limitations are noted: rigor of the epigenetics investigations is reduced by buccal cell use, the prior concern related to gene by environment correlations has not been fully addressed, resiliency measures are limited, and the role of the mothers' potential partners is insufficiently considered. In spite of a few minor limitations, this is a high impact study from an excellent team on the important public health topic of effects of maternal depression on mother and child.

DESCRIPTION (provided by applicant): Perinatal depression (PND) affects more than 400,000 mother-infant dyads in the US each year, with devastating consequences. Mothers with PND exhibit reduced sensitivity to infant needs, increasing infant risk for impaired emotional regulation and insecure attachment. These dysregulated interactions in the first year of life are associated with impaired cognitive and socioemotional development, including child psychopathology and impaired executive function (EF). Mothers who experience PND are more likely to have continuing or relapsing depression and anxiety disorders, conferring further risk. Nevertheless, despite exposure to PND, some dyads emerge intact. The long-term goal of this research is to identify the psychobiological underpinnings of resilience among mother-child dyads exposed to PND and longer-term maternal depression and anxiety trajectories (MDATs). The objectives of this proposal are to characterize MDAT heterogeneity during the first 5 years of the child's life, to identify mediators that explain the mechanisms through which MDATs influence child outcomes, and identify moderators that may serve as intervention points for promoting dyadic resilience. We will leverage an existing pool of participants in the Mood, Mother and Infant (MMI) study (R01HD073220, mmi.web.unc.edu, PI Stuebe), an ongoing longitudinal cohort study that we have led of mother-infant dyads (N=222) who have been extensively phenotyped during the first postpartum year. Our central hypothesis is that oxytocin plays a central role in dyadic development, indexed by associations between OT psychobiology, genetics and epigenetics and both MDATs and child development outcome. The rationale for this work is that our findings will inform targeted interventions to facilitate resilience and diminish the sequelae of maternal depression. We will accomplish the objective of our application by pursuing the following specific aims via an MMI follow-up study, the Mood, Mother and Child (MMC) study: 1) Elucidate the role of OT in the maternal psychobiological underpinnings of MDATs and parenting behavior, including effects of exogenous oxytocin (OT) on HPA axis reactivity; 2) Determine psychosocial mediators and moderators of associations between MDATs and child developmental outcome; and 3) Determine the extent to which child OXT and OXTR genotype moderates associations between MDATs, sensitivity, attachment quality, and developmental outcome; quantify the extent to which child epigenetic changes in OT and OXTR mediate associations between MDATs and developmental outcome. The expected outcomes of this work will be the determination of both predictive and protective factors for mother-infant dyads affected by depression and anxiety, laying the groundwork for novel approaches to promote resilience. Such results will have a positive impact by informing interventions to prevent intergenerational transmission of depression and anxiety.

PUBLIC HEALTH RELEVANCE: The proposed research is relevant to public health because it will identify the psychobiological underpinnings of resilience among mother-child dyads exposed to maternal depression, a common and morbid condition that impacts the health of more than 400,000 mother-infant dyads every year. The project is relevant to NICHD's mission because it will identify developmental factors associated with psychosocial adjustment among children exposed to maternal depression, ensuring that all children have the chance to achieve their full potential for healthy and productive lives.

CRITIQUE 1

Significance: 3
Investigator(s): 1
Innovation: 4
Approach: 4
Environment: 2

Overall Impact: The proposed study will build on an existing cohort of mother-infant dyads to track the relationship between maternal depression and infant outcomes, and identify the moderating and mediating factors that contribute to infant risk and resilience. The premise of the project is strong and the methodology is rigorous in most cases. Strengths of the application include a multileveled psychobiological approach, excellent statistical methods, and an innovative intransal oxytocin (OT) trial to probe HPA and OT system function during stress in mothers. Major limitations are largely centered on insufficient detail regarding genomics and epigenomics methods and interpretation. Whole genome approaches are proposed, although only a few genes will be interrogated. Buccal cell DNA methylation is proposed as potential mediating mechanism of infant outcomes, but can only model, not imitate neuronal epigenomics. Minor limitations that nonetheless diminish impact include inadequate consideration of maternal partner support as a confound of dyad characteristics, and remaining concerns of the role of gene-environment correlations on results.

1. Significance:

Strengths

- This is a highly significant area of research with great potential for identifying some of the neuroendocrine and behavioral mechanisms for the impact of maternal depression on child development.

Weaknesses

- The significance of the genomic aim is unclear. The mismatch between methodology and proposed analyses (i.e., whole genome approaches are proposed but analyses will only include a few genes) diminishes impact. The use of proxy tissue for neural development is necessary in human epigenetic studies, but more consideration must be given to the limits of interpretation of data collected from proxy tissue and impact on results.

2. Investigator(s):

Strengths

- This is a superb team of investigators, well positioned to carry out the majority of procedures proposed.
- The Principal Investigators are established investigators, with many publications in the areas of oxytocin, maternal psychopathology, and child development.

- An advisory team of senior investigators has been assembled, which enhances confidence of project success.
- An experienced postdoctoral fellow with five publications in epigenetics and bioinformatics and statistical geneticist to be named will conduct genomics statistical analyses.

Weaknesses

- No limitations noted.

3. Innovation:

Strengths

- The use of intranasal oxytocin to probe HPA and OT function in mothers is highly innovative.

Weaknesses

- The genetic and epigenetic approaches proposed do not maximize potential innovation.

4. Approach:

Strengths

- The sequalae of potential influences of maternal depression on child mental health are well-considered and operationalized.
- The use of multileveled measures of oxytocin function as potential moderators of mother-child relationships is excellent.
- The consideration of sex differences is a major strength.

Weaknesses

- Whole genome and epigenome methods do not seem warranted given the analyses focus on one system. The justification for buccal cell as a model of neurons is not strong and there is insufficient conceptualization of how data from proxy tissue will be interpreted.
- Gene-environment correlations still present a significant potential confound. The proposed method for addressing potential gene-environment correlations, using infant temperament as a proxy for genetic influences, is not sufficiently justified.
- A minor concern is that although the population is predominantly married women, there is no consideration in the methods or statistical analysis of a potential confound of maternal characteristics with availability or quality of partner support.
- Another minor concern is that resilience is defined as “better than expected” outcomes, but it is unclear how this will be statistically or conceptually defined within the population.

5. Environment:

Strengths

- This is a superb environment for the proposed studies, and feasibility is enhanced by the fact that this location has housed prior projects which provide the basis for the proposed work as well as many other collaborative and related studies.

Weaknesses

- The location and equipment for proposed sequencing and array work is not adequately described.

Protections for Human Subjects:

Acceptable Risks and/or Adequate Protections

Data and Safety Monitoring Plan (Applicable for Clinical Trials Only):

Acceptable

Inclusion of Women, Minorities and Children:

- Sex/Gender: Distribution justified scientifically
- Race/Ethnicity: Distribution justified scientifically
- For NIH-Defined Phase III trials, Plans for valid design and analysis:
- Inclusion/Exclusion of Children under 18: Including ages <18; justified scientifically

Vertebrate Animals:

Not Applicable (No Vertebrate Animals)

Biohazards:

Acceptable

Resource Sharing Plans:

Acceptable

Authentication of Key Biological and/or Chemical Resources:

Not Applicable (No Relevant Resources)

Budget and Period of Support:

Recommend as Requested

CRITIQUE 2

Significance: 2
Investigator(s): 1
Innovation: 1
Approach: 1
Environment: 1

Overall Impact: This is a well-prepared and documented application that does a good job of clarifying and addressing concerns raised in the prior reviews. The project leverages a comprehensive earlier project with repeated visits of over 200 maternal-infant pairs, oversampled for mood disorder, that characterized the individuals and the dyad on multiple dimensions. This 5-year follow-up extends the focus on oxytocin as the principal linchpin in dyadic development and, through affective, behavioral, epigenetic and related neurohormonal systems, might translate the disruption imposed by maternal mood disorders to dysregulated child development. The research team is excellent and has already conducted high caliber work which forms the substrate for this project. Figure 1 provides a clear visual description of the conceptual model and the manner in which each of the three aims feeds into it. The revision also considers the role of sex as a biological variable and although the existing literature yields contradictory findings regarding differential vulnerability to maternal mood disorders by child sex,

preliminary data provided from the parent study suggest that child sex does indeed modify associations between trajectories of maternal mood disorders and outcomes, as does variation in parenting behaviors. The revised application will explicitly test for sex effects, including in epigenetic markers. A prior concern regarding sociodemographic characteristics of the sample is discussed in Approach below, but the investigators also note that a follow-up study is planned for an existing, more diverse, cohort. The prior review noted that relatively few studies based on the parent project have been published to date. The investigators' response, that they are awaiting conclusion of data collection, reveals the double-edged sword that is inherent in the management of complex longitudinal studies in which each individual element (the still-face paradigm, for example) requires large amounts of processing time before data can even be input into a statistical database. Couple this with a never-ending stream of incoming new data, each source with its own challenges, and the problem becomes exponential. Thus although the goal of avoiding piecemeal publication is laudable, inadequate identification of stand-alone questions that can be answered by already collected data can lessen the impact of this rigorously executed and important work.

1. Significance:

Strengths

- In recent years, the role of the "4th trimester" has taken a back seat to research on purported fetal programming via biological mediation during pregnancy. However, postnatal depression translated through effects on maternal-infant interaction and other social factors has clear sequelae for development that is often overlooked despite accrued evidence since the 1980s. The proposed study will reinvigorate this important avenue of inquiry through the examination of the role of oxytocin as the driver of dyadic success in relation to maternal mood disorders using modern technologies and current understanding of underlying processes.
- A child's developmental repertoire at age 5 is greatly expanded beyond that reportable at the last observation period at 12 months and is also more predictive of important subsequent outcomes. This allows testing on more meaningful outcome parameters while leveraging with the original study will allow reach-back to the peripartum period.

Weaknesses

- The influence of perinatal depression on children is repeatedly described as "devastating" which is a bit of hyperbole. Although it can cast a wide net on offspring outcomes, many children manage to not be significantly affected which is in fact a point of this work – to document the factors that underlie resilience and evaluate the continuum of outcomes.

2. Investigator(s):

Strengths

- The Co-PIs reflect expertise in peripartum women and mood disorders (Steube) and developmental psychology within family systems (Mills-Koonce) and have previous experience working together on the existing R01 which forms the substrate for this project. Studies on the topic often are driven by expertise in one domain or the other; here the blended approach shows through the rigor with which both elements are represented in the existing and proposed work.
- The roles of a number of investigators listed are described as part of a "senior advisory team" without appreciable effort. Although this might be of concern in a new collaboration in a new field, the remarkable success of the prior study alleviates this as a concern and instead reveals the vibrant intellectual community within which this research is taking place.

Weaknesses

- No limitations were noted in the prior review and none are noted here.

3. Innovation:

Strengths

- The study will examine both the predictive and the protective factors that link PND to outcomes. Inclusion of the latter is often overlooked but it is key to developing successful interventions that can mitigate deleterious effects.
- The oxytocin challenge is a unique feature of the project because it is believed to be the first to evaluate this response in women with older children within this research framework and few psychobiological studies that employ exogenous oxytocin delivery in relation to a stressor challenge designed to activate the HPA axis have included women at all.

Weaknesses

- None noted.

4. Approach:

Strengths

- Although the study protocol imposes a high participant burden, participating women are described as identifying with the project's goals and enjoy the process. This resonates with those who implement longitudinal studies in this population and recognize that it suggests that the research team interacts with participants in a respectful fashion as partners. Retention/tracking is boosted by contacts in 6 month intervals for questionnaire completion. That the follow-up protocol for the proposed study is described as less intensive than the original further alleviates this concern.
- A prior concern related to the relatively homogenous nature of the sample population, particularly in terms of education level. Consistent with the investigators' response, unless there is reason to suspect that the underlying biobehavioral processes differ by race/SES as opposed to being moderated by contextual factors, this does not necessarily affect generalizability of the research question and eliminates the need to control for socioeconomic and related factors that can overwhelm the ability to detect associations. Practicalities of focusing on more educated women include higher rates of and more protracted duration of breastfeeding and higher participation in follow-up studies which contribute to study success.
- In response to the prior review, preliminary data showing differential findings by child sex have been presented and the consideration of child sex as a biological and social factor has been integrated into the current study.
- The inclusion of child temperament at the 5 year visit, and its integration into the analytic plan, is worthwhile.
- The rigor of the design allows evaluation of the role of oxytocin in terms of tonic, observational associations (both longitudinal and cross-sectional), phasic effects (response to stressor), a challenge model (placebo v oxytocin administration) as well as genetic/epigenetic variation. This is truly a remarkable undertaking.
- The protocols for women and children are comprehensive and reflect the state of the field but should not be overwhelming. The team is experienced in testing children. The physiological data collection systems afforded by MindWare provide strong measures of heart rate variability and PEP, yielding indications of parasympathetic and sympathetic activity.

Weaknesses

- Research on dyadic human systems is inherently complex. As a result, the investigators run the risk of generating too much complicated data without sufficient capacity to disseminate it in peer

reviewed journals, as opposed to abstracts. This can limit the impact of even the most well-supported premise and rigorously constructed research plan.

5. Environment:

Strengths

- UNC at both Chapel Hill and Greensboro have long-standing reputations for high quality research in all of the domains represented in this application. This includes a wealth of riches in development psychology at both campuses.
- The multidimensional nature of the project leverages significant infrastructure including a fully equipped laboratory for implementing experimental protocols and evaluating maternal and child behavioral and psychophysiological functioning.
- Qualified specialized services, including the Institutional Drug Service (IDS) for supporting the OT challenge, and the Biospecimen core for sample processing, are already in place and necessary for successful completion of the project

Weaknesses

- None noted

Protections for Human Subjects:

Acceptable Risks and/or Adequate Protections

- The study team is experienced in working with populations of women with mood disorders and their children. The plan as stated is comprehensive and appropriate.

Data and Safety Monitoring Plan (Applicable for Clinical Trials Only): appropriate

Inclusion of Women, Minorities and Children:

- Sex/Gender: Distribution justified scientifically
- Race/Ethnicity: Distribution justified scientifically
- For NIH-Defined Phase III trials, Plans for valid design and analysis:
- Inclusion/Exclusion of Children under 18: Including ages <18; justified scientifically
- The study includes 5 year old children, roughly equally distributed by sex, and their adult mothers. Based on the parent study that determines eligibility, the expected distribution by race/ethnicity is 79.5 percent white, 13 percent African American, 5 percent Asian; approximately 10 percent is of Hispanic ethnicity.

Vertebrate Animals:

Not Applicable (No Vertebrate Animals)

Biohazards:

Not Applicable (No Biohazards)

Resubmission:

- The application has done a thorough job of responding to the prior reviews. Features of the responses are integrated into the overall impact paragraph as well as other segments of this review.

Authentication of Key Biological and/or Chemical Resources:

Not Applicable (No Relevant Resources)

Budget and Period of Support:

Recommend as Requested

CRITIQUE 3

Significance: 3
Investigator(s): 1
Innovation: 2
Approach: 3
Environment: 1

Overall Impact: This project is addressing an important public health issue. It is a continuation and extension of a project focused on maternal depression and anxiety trajectories (MDAT) across the first 5 years of the child's life. The research team is very strong. They are collecting a very extensive set of mediating and moderating variables for their complex model. Their basic plan is to continue the follow up from their existing R01 on perinatal effects on infant outcomes at age 1 to age 5 years. The proposed study would continue following the cohort online at a 6 month intervals until the child's age of 5 years. The study burden appears intense, covering psychosocial, psychiatric, biological and genetic assessments. To date their follow up response rate has been very good suggesting that their continuing response rate should be reasonable. Their response to previous reviews is appropriate.

Protections for Human Subjects:

Acceptable Risks and/or Adequate Protections

- The human subjects plan appears well thought out

Data and Safety Monitoring Plan (Applicable for Clinical Trials Only):

Not Applicable (No Clinical Trials)

Inclusion of Women, Minorities and Children:

- Sex/Gender: Distribution justified scientifically
- Race/Ethnicity: Distribution not justified scientifically
- For NIH-Defined Phase III trials, Plans for valid design and analysis: Not applicable
- Inclusion/Exclusion of Children under 18: Including ages <18; justified scientifically
- The sample inclusion criteria are appropriate and have been revised in response to previous critique

Vertebrate Animals:

Not Applicable (No Vertebrate Animals)

Biohazards:

Not Applicable (No Biohazards)

Revision:

- They have responded well to previous critique

Authentication of Key Biological and/or Chemical Resources:

Not Applicable (No Relevant Resources)

Budget and Period of Support:

Recommend as Requested

THE FOLLOWING SECTIONS WERE PREPARED BY THE SCIENTIFIC REVIEW OFFICER TO SUMMARIZE THE OUTCOME OF DISCUSSIONS OF THE REVIEW COMMITTEE, OR REVIEWERS' WRITTEN CRITIQUES, ON THE FOLLOWING ISSUES:

PROTECTION OF HUMAN SUBJECTS: ACCEPTABLE

INCLUSION OF WOMEN PLAN: ACCEPTABLE
The study will enroll children regardless of sex and their adult mothers; this is scientifically justified.

INCLUSION OF MINORITIES PLAN: ACCEPTABLE
The expected distribution by race/ethnicity is 79.5 percent white, 13 percent African American, 5 percent Asian; approximately 10 percent is of Hispanic ethnicity; this is scientifically justified.

INCLUSION OF CHILDREN PLAN: ACCEPTABLE
The study will enroll children from infancy to five-years-old; this is scientifically justified.

COMMITTEE BUDGET RECOMMENDATIONS: The budget was recommended as requested.

Footnotes for 1 R01 HD093901-01A1; PI Name: Stuebe, Alison M

NIH has modified its policy regarding the receipt of resubmissions (amended applications). See Guide Notice NOT-OD-14-074 at http://grants.nih.gov/grants/guide/notice-files/NOT-OD-14-074.html. The impact/priority score is calculated after discussion of an application by averaging the overall scores (1-9) given by all voting reviewers on the committee and multiplying by 10. The criterion scores are submitted prior to the meeting by the individual reviewers assigned to an application, and are not discussed specifically at the review meeting or calculated into the overall impact score. Some applications also receive a percentile ranking. For details on the review process, see http://grants.nih.gov/grants/peer_review_process.htm#scoring.

MEETING ROSTER

Biobehavioral Mechanisms of Emotion, Stress and Health Study Section
Biobehavioral and Behavioral Processes Integrated Review Group
CENTER FOR SCIENTIFIC REVIEW
MESH
02/26/2018 - 02/27/2018

Notice of NIH Policy to All Applicants: Meeting rosters are provided for information purposes only. Applicant investigators and institutional officials must not communicate directly with study section members about an application before or after the review. Failure to observe this policy will create a serious breach of integrity in the peer review process, and may lead to actions outlined in NOT-OD-14-073 at https://grants.nih.gov/grants/guide/notice-files/NOT-OD-14-073.html and NOT-OD-15-106 at https://grants.nih.gov/grants/guide/notice-files/NOT-OD-15-106.html, including removal of the application from immediate review.

CHAIRPERSON(S)

HALL, MARTICA HELON, PHD
PROFESSOR
DEPARTMENT OF PSYCHIATRY, PSYCHOLOGY
AND CLINICAL AND TRANSLATIONAL SCIENCE
UNIVERSITY OF PITTSBURGH
PITTSBURGH, PA 15213

MEMBERS

ALBERT, MICHELLE A, MD, MPH
PROFESSOR
DIVISION OF CARDIOLOGY
DEPARTMENT OF MEDICINE
UNIVERSITY OF CALIFORNIA, SAN FRANCISCO
SAN FRANCISCO, CA 94143

BADR, M. SAFWAN, MD
PROFESSOR AND CHAIRMAN
DEPARTMENT OF INTERNAL MEDICINE
WAYNE STATE UNIVERSITY
DETROIT, MI 48201

BISHOP, JEFFREY R, PHARMD *
ASSOCIATE PROFESSOR
DEPARTMENT OF EXPERIMENTAL AND CLINICAL
PHARMACOLOGY
UNIVERSITY OF MINNESOTA
MINNEAPOLIS, MN 55455

BIXLER, EDWARD, PHD *
PROFESSOR
DEPARTMENT OF PSYCHIATRY
PENN STATE MILTON S. HERSHEY MEDICAL CENTER
HERSHEY, PA 17033

BLACKFORD, JENNIFER URBANO, PHD *
PROFESSOR
PSYCHIATRY AND BEHAVIORAL SCIENCES
VANDERBILT UNIVERSITY SCHOOL OF MEDICINE
VANDERBILT UNIVERSITY
NASHVILLE, TN 37212

BORTOLATO, MARCO, PHD
ASSOCIATE PROFESSOR
DEPARTMENT OF PHARMACOLOGY AND TOXICOLOGY
UNIVERSITY OF UTAH
SALT LAKE CITY, UT 84112

BROWN, E SHERWOOD, MD, PHD *
PROFESSOR
VICE CHAIRMAN FOR CLINICAL RESEARCH
DEPARTMENT OF PSYCHIATRY
UT SOUTHWESTERN MEDICAL CENTER
DALLAS, TX 75390

BURGESS, HELEN J, PHD
PROFESSOR
DEPARTMENT OF BEHAVIORAL SCIENCES
RUSH UNIVERSITY MEDICAL CENTER
CHICAGO, IL 60612

CONRADT, LIZ D, PHD *
ASSISTANT PROFESSOR
DEPARTMENT OF PSYCHOLOGY
UNIVERSITY OF UTAH
SALT LAKE CITY, UT 84103

DEVRIES, ANNE COURTNEY, PHD
PROFESSOR
DEPARTMENT OF MEDICINE
SECTION OF HEMATOLOGY AND ONCOLOGY
WEST VIRGINIA UNIVERSITY
MORGANTOWN, WV 26506

DI PIETRO, JANET A, PHD *
PROFESSOR
DEPARTMENT OF POPULATION, FAMILY
AND REPRODUCTIVE HEALTH
JOHNS HOPKINS BLOOMBERG SCHOOL OF PUBLIC HEALTH
BALTIMORE, MD 21205

DRURY, STACY SCHMIDT, MD, PHD
ASSOCIATE PROFESSOR
DEPARTMENT OF PSYCHIATRY AND BEHAVIORAL SCIENCES
SCHOOL OF MEDICINE
TULANE UNIVERSITY
NEW ORLEANS, LA 70112

FRIEDMAN, ELLIOT MICHAEL, PHD
BERNER HANLEY ASSOCIATE PROFESSOR
DEPARTMENT OF HUMAN DEVELOPMENT AND FAMILY STUDIES
PURDUE UNIVERSITY
WEST LAFAYETTE, IN 47907

FULIGNI, ANDREW J, PHD *
PROFESSOR IN-RESIDENCE
DEPARTMENT OF PSYCHIATRY
UNIVERSITY OF CALIFORNIA, LOS ANGELES
LOS ANGELES, CA 90024

HAROON, EBRAHIM, MD *
ASSISTANT PROFESSOR
DEPARTMENT OF PSYCHIATRY AND BEHAVIORAL SCIENCE
EMORY UNIVERSITY
ATLANTA, GA 30322

HONG, SUZI, PHD
ASSOCIATE PROFESSOR
DEPARTMENT OF PSYCHIATRY, FAMILY MEDICINE AND PUBLIC HEALTH
UNIVERSITY OF CALIFORNIA, SAN DIEGO
LA JOLLA, CA 92093

KEENAN, KATHRYN ELIZABETH, PHD *
PROFESSOR
DEPARTMENT OF PSYCHIATRY AND BEHAVIORAL NEUROSCIENCE
UNIVERSITY OF CHICAGO
CHICAGO, IL 60637

KINNALLY, ERIN LORAINE, PHD *
ASSISTANT PROFESSIONAL RESEARCHER
DEPARTMENT OF PSYCHOLOGY
UNIVERSITY OF CALIFORNIA, DAVIS
DAVIS, CA 95616

LAMBERT, SHARON F, PHD *
ASSOCIATE PROFESSOR
DEPARTMENT OF PSYCHOLOGY
GEORGE WASHINGTON UNIVERSITY
WASHINGTON, DC 20052

MUJAHID, MAHASIN S, PHD *
PROFESSOR AND DIRECTOR
SCHOOL OF PUBLIC HEALTH
UNIVERSITY OF CALIFORNIA, BERKELEY
BERKELEY, CA 94720

O'CONNOR, THOMAS G, PHD
PROFESSOR
DEPARTMENT OF PSYCHIATRY
UNIVERSITY OF ROCHESTER MEDICAL CENTER
ROCHESTER, NY 14642

ONG, JASON C, PHD
ASSOCIATE PROFESSOR
DEPARTMENT OF NEUROLOGY
FEINBERG SCHOOL OF MEDICINE
NORTHWESTERN UNIVERSITY
CHICAGO, IL 60611

RODRIGUEZ, CARLOS JOSE, MD
PROFESSOR
DIVISION OF PUBLIC HEALTH SCIENCES
DEPARTMENT OF EPIDEMIOLOGY AND PREVENTION
WAKE FOREST SCHOOL OF MEDICINE
WINSTON SALEM, NC 27157

SHANKMAN, STEWART AARON, PHD
PROFESSOR
DEPARTMENT OF PSYCHOLOGY
UNIVERSITY OF ILLINOIS AT CHICAGO
CHICAGO, IL 60607

SHIRTCLIFF, ELIZABETH ANNE, PHD *
ASSOCIATE PROFESSOR
DEPARTMENT OF HUMAN DEVELOPMENT AND FAMILY STUDIES
COLLEGE OF HUMAN SCIENCES
IOWA STATE UNIVERSITY
AMES , IA 50011

STARKWEATHER, ANGELA RENEE, PHD
PROFESSOR
CENTER FOR ADVANCEMENT IN MANAGING PAIN
SCHOOL OF NURSING
UNIVERSITY OF CONNECTICUT
STORRS, CT 06269

TONELLI, LEONARDO H, PHD
ASSOCIATE PROFESSOR
DEPARTMENT OF PSYCHIATRY
UNIVERSITY OF MARYLAND SCHOOL OF MEDICINE
BALTIMORE, MD 21201

WONG, MARIA M., PHD
PROFESSOR
DEPARTMENT OF PSYCHOLOGY
IDAHO STATE UNIVERSITY
POCATELLO, ID 83209

YOUNGSTEDT, SHAWN D, PHD
PROFESSOR
COLLEGE OF NURSING AND HEALTH INNOVATION
COLLEGE OF HEALTH SOLUTIONS
ARIZONA STATE UNIVERSITY
PHOENIX, AZ 85004

SCIENTIFIC REVIEW OFFICER

SMITH, SAMANTHA, PHD
SCIENTIFIC REVIEW OFFICER
CENTER FOR SCIENTIFIC REVIEW
NATIONAL INSTITUTES OF HEALTH
6701 ROCKLEDGE DRIVE ROOM 3170
BETHESDA, MD 20892

EXTRAMURAL SUPPORT ASSISTANT

ZIMBRO, CYNTHIA
LEAD EXTRAMURAL SUPPORT ASSISTANT
CENTER FOR SCIENTIFIC REVIEW
NATIONAL INSTITUTES OF HEALTH
BETHESDA, MD 20892

* Temporary Member. For grant applications, temporary members may participate in the entire meeting or may review only selected applications as needed.

Consultants are required to absent themselves from the room during the review of any application if their presence would constitute or appear to constitute a conflict of interest.
